# OPTIMISM, QUALITY OF LIFE, AND COGNITION IN RECENT NURSING HOME RESIDENTS

**DOI:** 10.1093/geroni/igz038.419

**Published:** 2019-11-08

**Authors:** Diana DiGasbarro, Kimberly Van Haitsma, Suzanne Meeks, Benjamin T Mast

**Affiliations:** 1 University of Louisville, Louisville, Kentucky, United States; 2 Penn State, University Park, Pennsylvania, United States

## Abstract

Dispositional optimism may be an important resource for well-being across the lifespan. However, the relationship between optimism and quality of life in recent nursing home residents with and without cognitive impairment has not been examined. The aim of this study is to fill this gap in a sample of 66 older adults with a mean age of 74.59 years old (SD=10.37) who were admitted to a nursing home within the previous 30 days. Sixty older adults completed measures of cognition, quality of life, and optimism, and thus were included in analysis for the current study. Participants were split into groups based on the presence or absence of cognitive impairment, and linear regressions were conducted to examine the relationship between optimism and quality of life. In recent nursing home residents without cognitive impairment (n=30), optimism did not predict quality of life and accounted for a very small amount of variance (R2=.042, p=.280). However, in recent nursing home residents with cognitive impairment (n=32), optimism accounted for 20.9% of the variance in quality of life (R2=.209, p=.009). Higher levels of optimism were associated with better quality of life. Future research should explore why a stable trait like dispositional optimism is a stronger predictor of quality of life in recent nursing home residents with cognitive impairment compared to those without cognitive impairment. This line of research would be synergistic with emerging research on the identification and encouragement of strengths in older adults with cognitive impairment.

